# Concordance among four commercially available, validated programmed cell death ligand-1 assays in urothelial carcinoma

**DOI:** 10.1186/s13000-019-0873-6

**Published:** 2019-09-02

**Authors:** Magdalena Zajac, Marietta Scott, Marianne Ratcliffe, Paul Scorer, Craig Barker, Hytham Al-Masri, Marlon C. Rebelatto, Jill Walker

**Affiliations:** 1 Oncology Companion Diagnostics Unit, Precision Medicine, R&D Oncology, AstraZeneca, Cambridge, UK; 2 Diagnostic Development Unit, Precision Medicine, R&D Oncology, AstraZeneca, Cambridge, UK; 3 Hematogenix, Tinley Park, IL USA; 4 MedImmune, Gaithersburg, MD USA

**Keywords:** PD-L1, Urothelial carcinoma, Assay, VENTANA PD-L1 SP263

## Abstract

**Background:**

Antibodies targeting the programmed cell death-1 (PD-1)/PD-ligand 1 (PD-1/PD-L1) checkpoint have shown promising clinical activity in patients with advanced urothelial carcinoma (UC). Expression of PD-L1 in UC tumors has been investigated using different antibody clones, staining protocols, and scoring algorithms. The aim was to establish the extent of concordance among PD-L1 immunohistochemistry (IHC) assays.

**Methods:**

Tumor biopsy samples (*N* = 335) were assessed using four commercially available PD-L1 assays: VENTANA SP263, VENTANA SP142, PD-L1 IHC 28–8 pharmDx, and PD-L1 IHC 22C3 pharmDx. PD-L1 analytical staining and classification concordance, including agreement between clinically relevant scoring algorithms, were investigated using overall/positive/negative percentage agreement (OPA/PPA/NPA).

**Results:**

Good analytical correlation was observed among the VENTANA SP263, PD-L1 IHC 22C3 pharmDx, and PD-L1 IHC 28–8 pharmDx assays for tumor cell (TC) and immune cell (IC) PD-L1 staining with Spearman rank coefficients of 0.92–0.93 for TCs and 0.88–0.91 for ICs. However, concordance (preset criterion: ≥85%) between patient PD-L1 status when applying the TC or IC_ICArea_ ≥ 25% (VENTANA SP263) cutoff was only achieved for PD-L1 IHC 22C3 pharmDx versus VENTANA SP263 (OPA 92.2%, PPA 86.4%, NPA 95.4%). Differences were observed between patient populations with UC tumors classified as PD-L1 high versus PD-L1 low/negative using combined positive score (CPS) ≥1, CPS ≥10, IC ≥5%, and TC/IC ≥25%.

**Conclusions:**

The VENTANA SP263 and PD-L1 IHC 22C3 pharmDx assays are analytically similar in UC. When the different PD-L1 assays were combined with their specified clinical scoring algorithms, differences were seen in patient classification driven by substantial differences in scoring approaches.

**Electronic supplementary material:**

The online version of this article (10.1186/s13000-019-0873-6) contains supplementary material, which is available to authorized users.

## Background

**Fig. 1:**
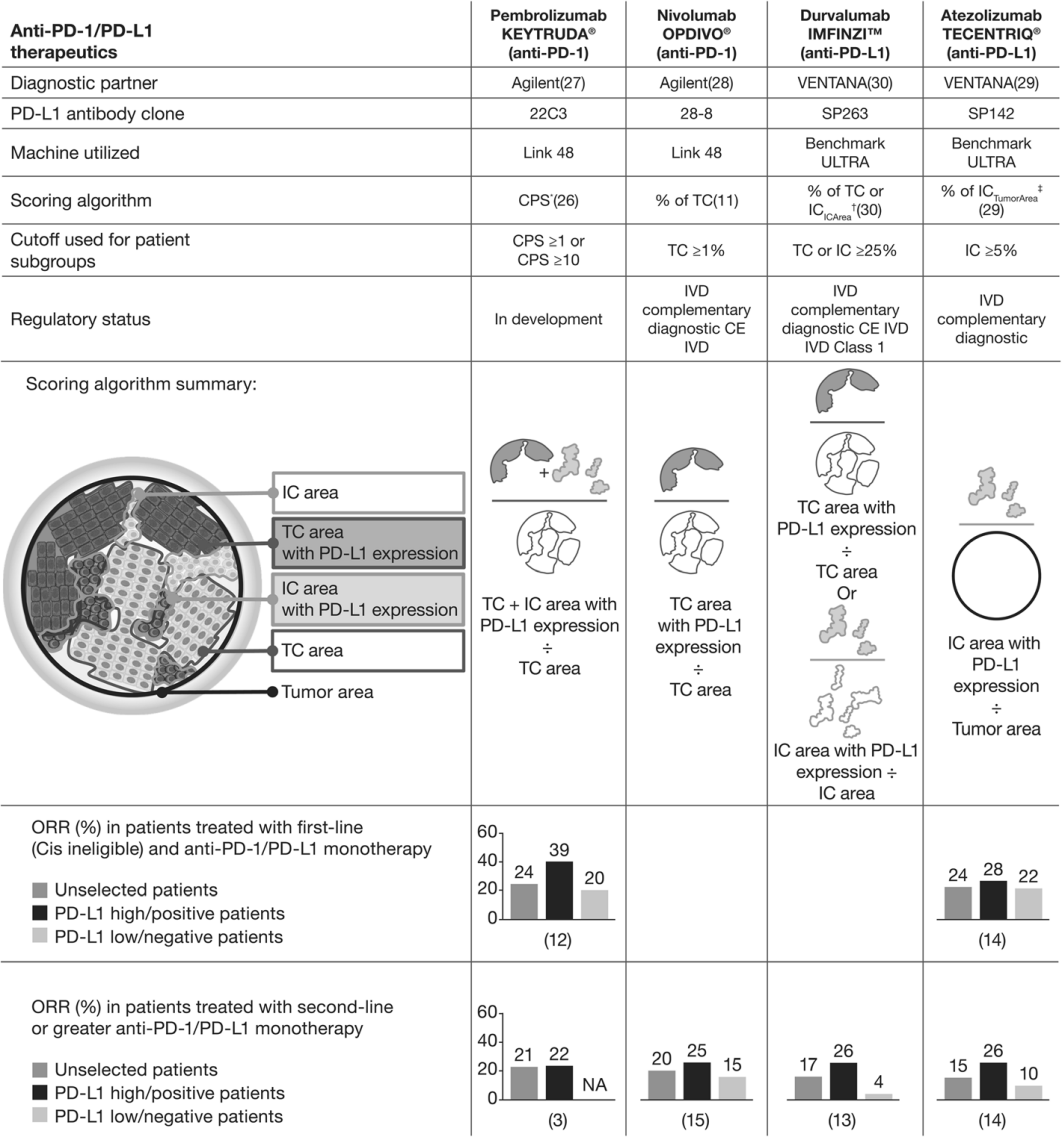
Comparison of PD-L1 assays for UC and differences in immune cell measurement and scoring algorithm. *Ratio of tumor cells (TC) and immune cells (IC) relative to number of all TC. ^†^IC score is the percentage area of ICs present exhibiting PD-L1 positive IC staining. ^‡^IC score is the proportion of ICs that are PD-L1 positive, expressed in relation to tumor area. *CE* European Conformity, *Cis* cisplatin, *IVD* in vitro diagnostic

Anti-programmed cell death ligand-1 (PD-L1; durvalumab, atezolizumab, and avelumab) and anti-programmed death-1 (PD-1; nivolumab and pembrolizumab) antibodies have shown promising clinical activity in patients with advanced urothelial carcinoma (UC) [1–12]. These agents are approved by the US Food and Drug Administration for the treatment of patients with locally advanced or metastatic UC with disease progression during or following platinum-containing chemotherapy or disease progression within 12 months of neoadjuvant or adjuvant treatment with platinum-containing chemotherapy. Nivolumab, pembrolizumab, and atezolizumab are also approved in this indication by the European Medicines Agency [13–20]. Pembrolizumab and atezolizumab have also received accelerated approval for first-line treatment of locally advanced or metastatic UC in patients ineligible for cisplatin-containing chemotherapy. Higher clinical response rates with these agents tend to be observed in patients with UC tumors with high PD-L1 expression than in those with tumors with PD-L1 low/negative expression, with correlation seen between overall response rate and PD-L1 expression in patients treated with first-line (cisplatin-ineligible) and second-line or greater anti–PD-1/PD-L1 monotherapy in UC (Fig. 1) [1, 4, 5, 7–9, 11]. PD-L1 expression levels in UC tumors may help physicians identify patients who are more likely to benefit from an anti–PD-1/PD-L1 therapy.

Four validated, commercially available assays (VENTANA PD-L1 SP263 and VENTANA PD-L1 SP142 [Ventana Medical Systems, Inc., Tucson, Arizona, USA], and PD-L1 immunohistochemistry [IHC] 22C3 pharmDx and PD-L1 IHC 28–8 pharmDx [Agilent Technologies, Santa Clara, California, USA]) have been developed independently in conjunction with immunotherapies targeting the PD-1/PD-L1 pathway. These assays use different antibodies, IHC protocols, scoring algorithms, and cutoffs to define high/low PD-L1 expression in UC (Fig. 1) [21–30]. Unlike the application of these assays in non–small cell lung cancer (NSCLC), in UC, the PD-L1 scoring approaches differ widely among the various assays. In UC, VENTANA SP142 assesses the proportion of tumor area occupied by PD-L1-stained immune cells (IC) (% of IC_TumorArea_), while VENTANA SP263 utilizes the proportion of ICs with PD-L1 staining as a proportion of the IC area as well as the proportion of tumor cells (TCs) with PD-L1 membrane staining (% of TC or IC_ICArea_) (Fig. 1). PD-L1 IHC 22C3 pharmDx uses the combined positive score (CPS) of TCs and ICs with PD-L1 staining, while PD-L1 IHC 28–8 pharmDx measures the proportion of TCs with PD-L1 membrane staining only (% of TC) (Fig. 1). In addition to the difference in scoring methods between the assays, in UC, there are significant differences between assays in the cutoffs used to define PD-L1 expression level [24, 27–31]. These differences raise the question of whether the UC patient populations defined as PD-L1 high are the same across clinical trials based on the algorithms (particular combination of scoring method and cutoff) used, and therefore, whether results can be compared across trials.

To conserve patient tissue and pathology resources, the use of a single PD-L1 assay for tumor testing is desirable. However, such harmonization requires a thorough understanding of the concordance between staining, scoring algorithms, and cutoffs. To enable this, and to demonstrate interchangeable use, a first step is to compare the analytical performance of the available assays. Good analytical concordance has been previously demonstrated among three validated, commercially available PD-L1 IHC assays (VENTANA SP263, PD-L1 IHC 22C3 pharmDx, and PD-L1 IHC 28–8 pharmDx) across multiple TC PD-L1 protein expression cutoffs using samples from patients with NSCLC [31] or head and neck squamous cell carcinoma (HNSCC) [24]. The VENTANA SP142 assay was also evaluated, but did not show good concordance with the other three assays for TCs, an observation that has been supported across multiple independent studies [32, 33]. More recently, the analytical comparability of these four assays has been investigated in staining of a small number of samples from patients with advanced UC for IC and TC staining, showing comparable results across assays, except for significantly lower staining of TC by VENTANA SP142; however, this was conducted in a small number of samples and no formal statistical evaluation was performed [34].

In addition to assessing the analytical performance of the four commercially available PD-L1 IHC tests, this study assessed the overlap between patient populations selected by these assays when different algorithms are used to define high versus low/negative PD-L1 expression. Comparing the technical performance of different assays and algorithms will allow appropriate interpretation of clinical outcomes for patients with UC treated with different anti–PD-1/PD-L1 therapies.

## Methods

### Study design

Archival formalin-fixed, paraffin-embedded clinical UC tumor sample blocks aged ≤5 years were obtained from commercial sources (Avaden BioSciences, Seattle WA, USA; Asterand Bioscience, Royston, UK; BioIVT, West Sussex, UK). AstraZeneca has a governance framework and processes to ensure that commercial sources have appropriate patient consent and ethical approval in place for collection of the samples for research purposes including use by for-profit companies.

Consecutive sections derived from tumor blocks were stained with VENTANA SP263, VENTANA SP142, PD-L1 IHC 22C3 pharmDx, and PD-L1 IHC 28–8 pharmDx according to their validated protocols for investigational use, and PD-L1 testing was carried out at Hematogenix (Tinley Park, IL, USA). The PD-L1 antibody clone (PD-L1 IHC 73–10 pharmDx), assessed in Blueprint phase II NSCLC and in metastatic breast cancer, was not commercially available at the time of analysis [35, 36]. A single pathologist, trained by the manufacturers (Clinical Laboratory Improvement Amendments program-certified laboratory, Hematogenix), scored all samples in a blinded fashion, which were batched on an assay-by-assay basis. There was a washout period of ≥0.5 days between scoring the different assays. A single pathologist was used to remove reader subjectivity as a factor, thus ensuring a true inter-assay comparison.

The following parameters were recorded for each case and each assay: percentage of TCs with membrane staining for PD-L1 (TC score), percentage of tumor area occupied by tumor infiltrating ICs (IC area), percentage of ICs staining for PD-L1 (IC_ICArea_ score) (as would be assessed for the VENTANA SP263 assay), percentage of tumor area occupied by PD-L1 staining tumor infiltrating ICs (IC_TumorArea_ score) (as would be assessed for the VENTANA SP142 assay), and CPS of the number of PD-L1 positive cells divided by the total number of TCs × 100 (as would be assessed for the PD-L1 IHC 22C3 pharmDX assay). TC, IC area, and IC_ICArea_ scores were recorded in 1% increments between 0 and 5%, and in 5% increments thereafter; IC_TumorArea_ score was scored in 1% increments; and CPS was scored in increments of 1.

### Comparison of scored and derived parameters

To determine whether it is possible to use derived values for IC_TumorArea_ and CPS, rather than scoring directly, derived parameters were calculated as follows:

$$ \bullet \mathrm{Derived}\ {\mathrm{IC}}_{\mathrm{TumorArea}}={\mathrm{IC}}_{\mathrm{IC}\mathrm{Area}}\ \mathrm{score}\times \mathrm{IC}\ \mathrm{area}. $$

$$ \bullet \mathrm{Derived}\ \mathrm{CPS}=\mathrm{TC}\ \mathrm{score}+\left({\mathrm{IC}}_{\mathrm{IC}\mathrm{Area}}\times \mathrm{IC}\ \mathrm{area}\right)/\left(1-\mathrm{IC}\ \mathrm{area}\right). $$

### Statistical analysis

#### Analytical concordance between assays

To assess the similarity in staining and scoring between the four assays, bubble plots and Spearman rank correlation coefficients were generated pairwise between assays for the TC, IC_ICArea_, and IC_TumorArea_ scores. Correlation was classed as “good” where ρ ≥ 0.85. Concordance between the scored and derived values for IC_TumorArea_ and CPS were assessed for the VENTANA SP142 and PD-L1 IHC 22C3 pharmDx assays, respectively, using the same approach. Plots showing the TC and IC_ICArea_ scores for each assay ranked by the average value across assays were also generated. To demonstrate similarity between assays without the influence of cases where both assays were scored at 0% for the given parameter, Spearman rank correlation coefficients (ρ) were also generated excluding these cases.

#### Clinical concordance between assays

Two aspects of clinical concordance were assessed: first, whether the VENTANA SP263 clinically relevant algorithm (25%TC/IC) selects the same patients when applied to different assays. To do this, for each of the four assays, the patient status was determined using the clinically relevant algorithm for the assay itself (Fig. 1) and also using the VENTANA SP263 algorithm (patient positive if either TC or IC_ICArea_ score is ≥25%).

Secondly, it was assessed whether the clinically relevant algorithms applied to the respective assays select the same patient population. Overall percentage agreement (OPA), negative percentage agreement (NPA), and positive percentage agreement (PPA) were calculated pairwise between assays using the appropriate comparator as reference assays for each clinically relevant cutoff; assays were considered concordant if OPA, PPA, and NPA were ≥ 85%. For each metric, the lower boundary of 95% confidence interval (CI) was calculated excluding upper bound using Clopper–Pearson method [37].

## Results

UC tumor samples ≤5 years old from a total of 335 patients were included in this analysis. Patient demographics are shown in Table 1. Approximately 75% of patients were aged > 65 years, and patients were predominantly male (72%). The majority of tumor samples were of urothelial carcinoma (98%) and 76% were invasive (stage II or higher). Most of the samples were from transurethral resection of the bladder tumor (70%).

**Table 1:** Patient demographics and baseline characteristics for study samples ≤5 years old

Demographic	Cases (*N* = 335)
Ethnicity	
African American	4.8%
Caucasian	39.7%
Other	8.4%
Unknown	47.2%
Age at surgery, years	
≤65	25.1%
>65	74.9%
Sex	
Female	27.8%
Male	72.2%
Histology	
Non-urothelial carcinoma^a^	3.3%
Urothelial carcinoma	97.6%
Stage	
0a	10.1%
I	13.7%
II	37.0%
III	31.0%
IIIB	0.3%
IV	7.8%
Grade	
High	77.7%
Low	22.1%
Unknown	0.3%
Sample type	
Biopsy	0.3%
Cystectomy	29.2%
TURBT	69.6%
Unknown	0.9%

^**a**^ Non-urothelial bladder cancer samples included in the analysis are further defined in Additional file 1

*TURBT* transurethral resection of bladder tumor

### Direct scoring versus derived scoring

For both IC_TumorArea_ and CPS, the correlation between the scored and derived scores, and the correlation between the ranks of the scored and derived scores, showed a high level of agreement (Spearman’s correlation coefficient of 0.997 and 0.999, respectively) (Additional file 2 for VENTANA SP142 and PD-L1 IHC 22C3 pharmDx). Therefore, scored and derived IC_TumorArea_ (and scored and derived CPS) can be considered interchangeable for each of these assays.

### Comparison of PD-L1 TC and IC staining

**Fig. 2:**
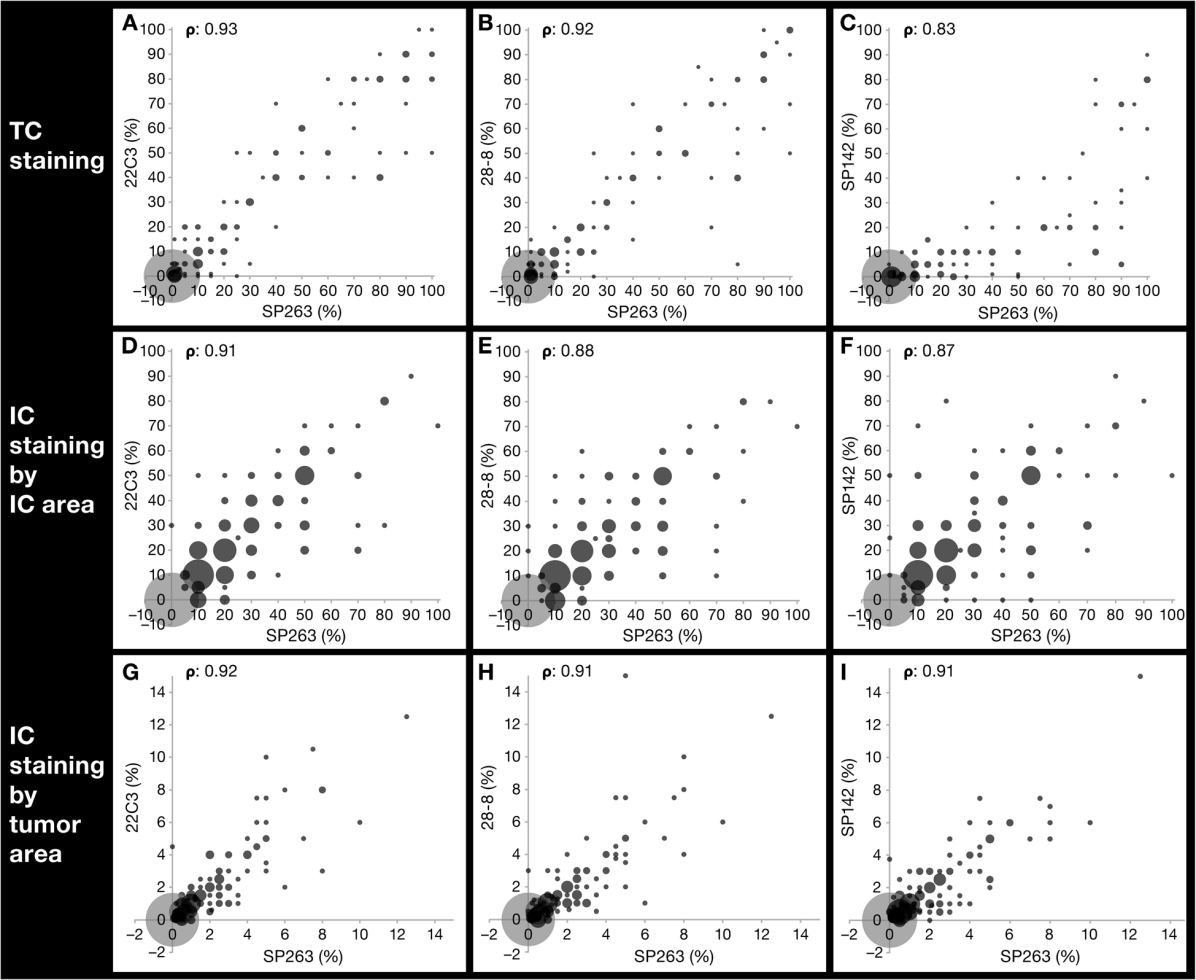
Comparison between PD-L1 IHC 22C3 pharmDx, PD-L1 IHC 28–8 pharmDx and VENTANA SP142 versus VENTANA SP263. Pairwise comparison between PD-L1 IHC 22C3 pharmDx (**a**, **d**, **g**), PD-L1 IHC 28–8 pharmDx (**b**, **e**, **h**) and VENTANA SP142 (**c**, **f**, **i**) assays versus VENTANA SP263 for TC and IC PD-L1 staining and their corresponding pairwise Spearman rank correlation coefficients (ρ). Pairwise Spearman rank correlation coefficients without zeros were 0.90, 0.89, and 0.82 for tumor cell (TC) staining; 0.77, 0.73, and 0.65 for immune cell (IC) staining by IC area; and 0.85, 0.83, and 0.80 for IC staining by tumor area for PD-L1 IHC 22C3 pharmDx, PD-L1 IHC 28–8 pharmDx, and VENTANA SP142 versus VENTANA SP263, respectively. *IC* immune cell, *IHC* immunohistochemistry, *PD-L1* programmed cell death ligand-1, *TC* tumor cell

There was a good linear analytical association between PD-L1 IHC 22C3 pharmDx and PD-L1 IHC 28–8 pharmDx assays and VENTANA SP263, for both TC and IC scores, with a good correlation between rank order of samples (ρ ≥ 0.85 for all cases) (Fig. 2). However, VENTANA SP142 had a poorer, nonlinear, correlation with VENTANA SP263 for TC (ρ = 0.83) (Fig. 2c), but a good linear correlation for IC (ρ = 0.87 and ρ = 0.91) (Fig. 2f and i).

PD-L1 IHC 22C3 pharmDx, PD-L1 IHC 28–8 pharmDx, and VENTANA SP263 assays showed similar PD-L1 prevalence for both TC and IC PD-L1 staining, with prevalence at the ≥25% cutoff ranging from 15.2 to 17.9% for TC staining, 21.5–25.1% for IC staining by IC area, and all at 0.6% for IC staining by tumor area (Additional files 2, 3, and 4).

VENTANA SP142 showed similar prevalence versus the other three assays for ICs (22.7% staining for IC by IC area; 0.3% for IC staining by tumor area), but was less sensitive for PD-L1 staining on TCs (prevalence 6.3% at the ≥25% cutoff) (Additional files 2, 3, and 4).

**Fig. 3:**
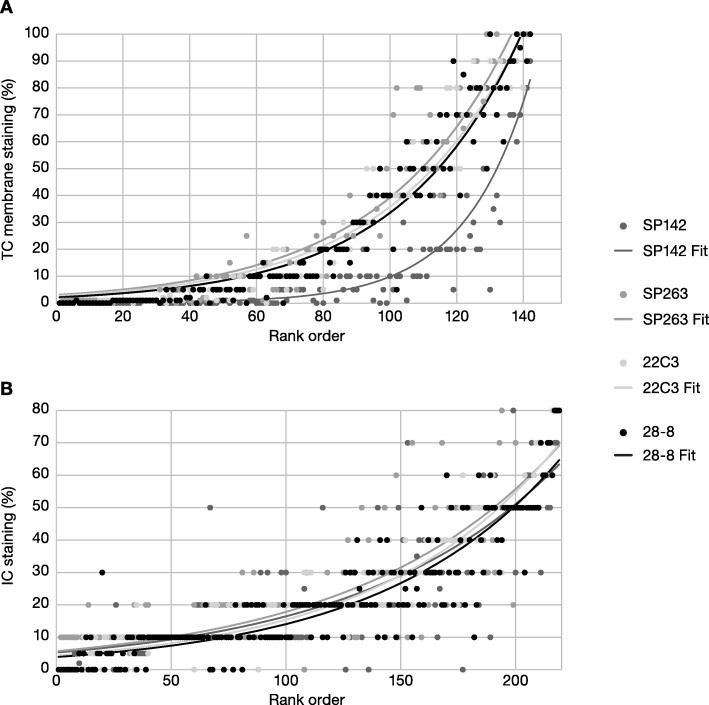
Percentage of PD-L1–positive TC and IC (per IC area) ranked by average value. Exponential fits to the data are provided for ease of visualization. **a** % of TC membrane staining ranked by average % of TC membrane staining. **b** % of IC membrane staining ranked by average % of IC membrane staining. *IC* immune cell, *PD-L1* programmed cell death ligand-1, *TC* tumor cell

The percentages of TC staining for PD-L1, ranked by average value, were similar for the PD-L1 IHC 22C3 pharmDx, PD-L1 IHC 28–8 pharmDx, and VENTANA SP263 assays, but lower for VENTANA SP142 (Fig. 3). However, the percentage of IC (per IC area) staining for PD-L1 was similar across all four assays (Fig. 3).

### Assay concordance at the VENTANA SP263 clinically relevant algorithm for UC

Agreement between patient PD-L1 status, as determined for each assay when applying the TC or IC_ICArea_ ≥ 25% cutoff (the algorithm validated for durvalumab patient selection, VENTANA SP263), was assessed using OPA, PPA, and NPA, setting the VENTANA SP263 assay as the reference (Table 2). OPA, PPA, and NPA of ≥85%, the preset criterion for assay concordance, were only achieved for PD-L1 IHC 22C3 pharmDx (OPA 92.2%, PPA 86.4%, NPA 95.4%). PD-L1 IHC 28–8 pharmDx and VENTANA SP142 had OPAs and NPAs ≥85% (OPA 90.7 and 85.7%; NPA 96.8 and 97.2%, respectively), but PPAs < 85% (79.7 and 64.4%, respectively) (Table 2), indicating that these two assays would select fewer patients as PD-L1 high compared with the reference assay. Results were similar in the two hundred fifty-five patients that had invasive disease (Additional file 5).

**Table 2:** OPA, PPA, and NPA between PD-L1 assays

Clinical algorithm	Comparator assay	VENTANA SP263 (TC/IC 25%) assay used as reference, % agreement (95% CI) ^a^		
		OPA	PPA	NPA
TC or IC_ICArea_ ≥ 25% (VENTANA SP263)	PD-L1 IHC 22C3 pharmDx	92.2% (89.4%)	86.4% (80.1%)	95.4% (92.3%)
	PD-L1 IHC 28–8 pharmDx	90.7% (87.7%)	79.7% (72.6%)	96.8% (94.0%)
	VENTANA SP142	85.7% (82.1%)	64.4% (56.5%)	97.2% (94.6%)
CPS ≥1 (PD-L1 IHC 22C3 pharmDx)	PD-L1 IHC 22C3 pharmDx	77.0% (72.9%)	90.7% (85.0%)	69.6% (64.0%)
CPS ≥10 (PD-L1 IHC 22C3 pharmDx)	PD-L1 IHC 22C3 pharmDx	81.5% (77.6%)	62.7% (54.8%)	91.7% (87.9%)
TC ≥1% (PD-L1 IHC 28–8 pharmDx)	PD-L1 IHC 28–8 pharmDx	75.5% (71.3%)	66.9% (59.1%)	80.2% (75.2%)
IC_TumorArea_ ≥ 5% (VENTANA SP142)	VENTANA SP142	69.9% (65.5%)	15.3% (10.1%)	99.5% (97.8%)

^a^ For each metric, lower boundary of 95% confidence interval (CI) was calculated with no upper bound using the Clopper–Pearson method [37]

*CPS* combined positive score, *IC* immune cell, *IHC* immunohistochemistry, *NPA* negative percentage agreement, *OPA* overall percentage agreement, *PD-L1* programmed cell death ligand-1, *PPA* positive percentage agreement, *TC* tumor cell

### Assay concordance using assay-specific clinically relevant algorithm for UC

When agreement was assessed between VENTANA SP263 and the other assays using the clinical algorithm validated for each individual assay (eg, IC_TumorArea_ ≥ 5% for VENTANA SP142), the criterion for assay concordance was not met for any of the three assays; although NPA values of 91.7 and 99.5% were found for PD-L1 IHC 22C3 pharmDx (CPS ≥10) and VENTANA SP142, respectively, with a PPA of 90.7% for PD-L1 IHC 22C3 pharmDx (CPS ≥1). NPA for PD-L1 IHC 28–8 pharmDx and PD-L1 IHC 22C3 pharmDx (CPS ≥1) were lower at 80.2 and 69.6%, respectively; all OPA and other PPA values were also < 85% (Table 2). This was also the case in the subset of patients with muscle-invasive disease (Additional file 5).

**Fig. 4:**
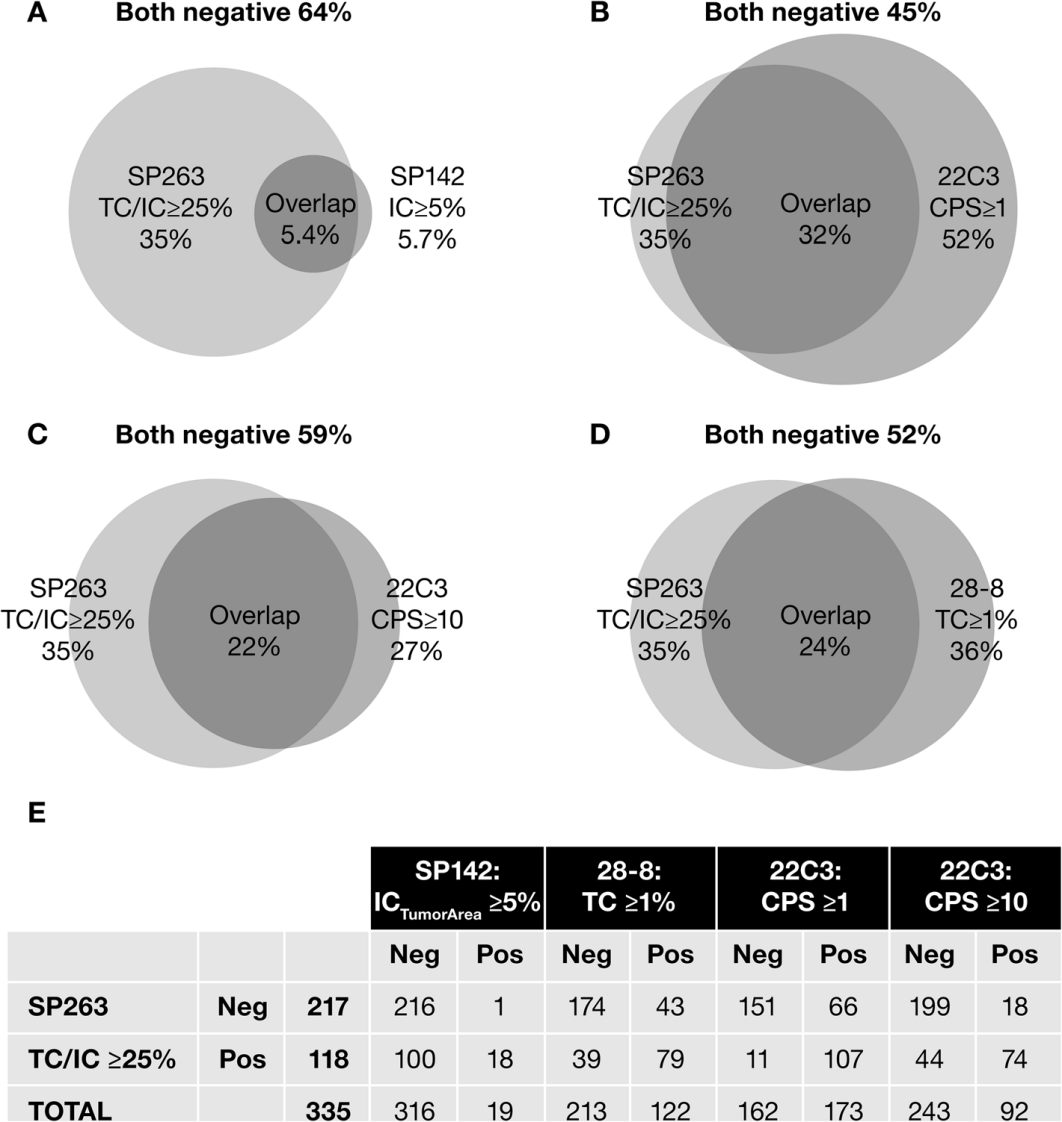
Differences in the populations selected by the investigated algorithms. **a** SP263 (TC/IC ≥25%) versus SP142 (IC_TumorArea_ ≥ 5%), **b** PD-L1 IHC 22C3 pharmDx (CPS ≥1), **c** PD-L1 IHC 22C3 pharmDx (CPS ≥10), and **d** PD-L1 IHC 28–8 pharmDx (TC ≥1%) algorithms, with subject numbers shown in **e**. *CPS* combined positive score, *IC* immune cell, *IHC* immunohistochemistry, *PD-L1* programmed cell death ligand-1, *TC* tumor cell

Differences between the populations selected by VENTANA SP263 versus VENTANA SP142, PD-L1 IHC 28–8 pharmDx, and PD-L1 IHC 22C3 pharmDx assays are illustrated in Fig. 4. Of 118 patients with tumors classified as PD-L1 high using the VENTANA SP263 algorithm, only 15.3% were also classified as PD-L1 high (PPA) using the VENTANA SP142 algorithm, while 66.9% were also classified as PD-L1 high using the PD-L1 IHC 28–8 pharmDx algorithm, 62.7% using the PD-L1 IHC 22C3 pharmDx at the CPS ≥10 cutoff, and 90.7% at the CPS ≥1 cutoff (Fig. 4). Of 217 patients with tumors classified as PD-L1 low/negative using the VENTANA SP263 algorithm, 99.5% were also classified as PD-L1 low/negative with the VENTANA SP142 algorithm, 80.2% with PD-L1 IHC 28–8 pharmDx, and 91.7% with PD-L1 IHC 22C3 pharmDx (Fig. 4).

## Discussion

In this study of 335 UC tumor samples, a high level of analytical concordance was observed among the VENTANA SP263, PD-L1 IHC 22C3 pharmDx, and PD-L1 IHC 28–8 pharmDx assays for TC and IC staining of PD-L1. Concordance criteria were met between categorization of patients using PD-L1 IHC 22C3 pharmDx and VENTANA SP263 using the TC/IC algorithm, suggesting that these assays could be used interchangeably in UC to determine PD-L1 expression levels. Importantly, this finding was also true in the subset of samples from patients with muscle invasive cancer. Despite the good rank correlation between assays, the other assays did not meet the agreement criteria, driven by lower PPA, suggesting a lower sensitivity for PD-L1 IHC 28–8 pharmDx, and particularly for VENTANA SP142.

Significant differences were observed between VENTANA SP142 and the other three assays for TC staining, whereas IC staining was similar. The analytical findings of our study are consistent with previously reported Blueprint observations in NSCLC [38] and other studies in NSCLC and HNSCC, where VENTANA SP142 also consistently detects fewer TCs [32, 33]. Our data also confirm the results of a recent study on samples from 30 patients with UC, which showed comparable results across assays for IC and TC staining, but significantly lower staining of TC by VENTANA SP142 [34].

Our study identified differences in the patient populations that would be classified as PD-L1 high versus PD-L1 low/negative by the PD-L1 IHC 22C3 pharmDx (CPS ≥1 and ≥ 10), VENTANA SP142 (IC ≥5%), and VENTANA SP263 (TC/IC ≥25%) algorithms. There was greater overlap between patient populations identified by VENTANA SP263 (TC/IC ≥25%) and PD-L1 IHC 22C3 pharmDx (CPS ≥1 and ≥ 10) than between VENTANA SP263 (TC/IC ≥25%) and VENTANA SP142 (IC ≥5%). According to our study, using the VENTANA SP142 assay and the IC ≥5% algorithm would misclassify a significant proportion of patients with UC tumors that are PD-L1 high according to the VENTANA SP263 assay (using the TC/IC algorithm); indeed, significantly fewer PD-L1 high patients would be identified using the VENTANA SP142 assay and algorithm. The discordance between patient populations may be explained by the inclusion of TCs in the VENTANA SP263 algorithm versus the VENTANA SP142 algorithm, the lower TC cutoffs for the PD-L1 IHC 22C3 pharmDx CPS algorithms versus the VENTANA SP263 algorithm, or the use of different denominators for the IC scoring approach in all three cases. Differences observed in assay sensitivity in this setting, particularly for PD-L1 IHC 28–8 pharmDx and VENTANA SP142, may also account for some variation in these patient populations. These differences in classification of patients as PD-L1 high versus PD-L1 low/negative using different assays and different algorithms suggest that caution should be taken when comparing clinical outcomes across studies.

Numerous assay compatibility initiatives aimed at reducing complexity in PD-L1 testing are ongoing [24, 38, 39]. In UC, a recent study showed good correlation between 22C3, E1L3N, and 28–8 assays for tumor cell PD-L1 staining, with SP142 showing lower sensitivity [40]. Another study showed excellent correlation between PD-L1 IHC 22C3 pharmDx, PD-L1 IHC 28–8 pharmDx, and E1L3N for tumor cell PD-L1 scoring, with lower reproducibility for IC scoring [41]. These data are consistent with the results presented here.

## Conclusions

These findings inform comparisons between studies using different PD-L1 tests, as well as the next steps toward harmonization of PD-L1 diagnostic testing in UC. With compelling assay concordance data, a single PD-L1 base assay could potentially be used for different therapies, but the appropriate, clinically validated algorithm must be applied to retain the connection between the cutoff and the therapy, and ideally this would be tested and confirmed in a prospective cohort. While the PD-L1 IHC 22C3 pharmDx and VENTANA SP263 assays could be used interchangeably, the appropriate, clinically validated algorithm for each therapy must be applied, eg, CPS for pembrolizumab and TC/IC for durvalumab.

## Additional files

Additional file 1: Non-urothelial bladder cancer samples included in the analysis (DOCX 14 kb)

Additional file 2: Plots for derived versus scored staining for VENTANA SP142 immune cell scores (A) and PD-L1 IHC 22C3 pharmDx CPS (B). *IC* immune cells, *CPS* combined positive score (TIF 1582 kb)

Additional file 3: PD-L1 proportion of samples above cutoff by assay, cutoff, and scored compartment. SP142 detects fewer TC PD-L1 positive cells than other PD-L1 assays. *IC* immune cells, *PD-L1* programmed cell death ligand-1, *TC* tumor cells (DOCX 15 kb)

Additional file 4: PD-L1 staining for four PD-L1 assays. *IC* immune cells, *TC* tumor cells (TIF 22699 kb)

Additional file 5: OPA, PPA, and NPA between PD-L1 assays in invasive UC samples (*N* = 255) (DOCX 16 kb)

## Data Availability

Data underlying the findings described in this manuscript may be obtained in accordance with AstraZeneca’s data sharing policy described at https://astrazenecagrouptrials.pharmacm.com/ST/Submission/Disclosure***.***

## Abbreviations

CE: European Conformity
CI: Confidence interval
Cis: Cisplatin
CPS: Combined positive score
HNSCC: Head and neck squamous cell carcinoma
IC: Immune cell
IC_ICArea,_: The proportion of immune cells with PD-L1 staining as a proportion of the immune cell area
IC_TumorArea,_: The proportion of tumor area occupied by PD-L1-stained immune cells
IHC: Immunohistochemistry
IVD: In vitro diagnostic
NPA: Negative percentage agreement
NREC: National Research Ethics Service Committee
NSCLC: Non–small cell lung cancer
OPA: Overall percentage agreement
PD-1: Programmed cell death-1
PD-L1: Programmed cell death ligand-1
PPA: Positive percentage agreement
RTB: Research Tissue Bank
TC: Tumor cell
TURBT: Transurethral resection of bladder tumor
UC: Urothelial carcinoma

## References

[1] Balar AV, Galsky MD, Rosenberg JE, Powles T, Petrylak DP, Bellmunt J (2017). Atezolizumab as first-line treatment in cisplatin-ineligible patients with locally advanced and metastatic urothelial carcinoma: a single-arm, multicentre, phase 2 trial. Lancet.

[2] Balar AV, Castellano DE, O'Donnell PH, Grivas P, Vuky J, Powles T (2017). Pembrolizumab as first-line therapy in cisplatin-ineligible advanced urothelial cancer: results from the total KEYNOTE-052 study population. J Clin Oncol.

[3] Bellmunt J, de Wit R, Vaughn DJ, Fradet Y, Lee JL, Fong L (2017). Pembrolizumab as second-line therapy for advanced urothelial carcinoma. N Engl J Med.

[4] Loriot Y, Rosenberg JE, Powles TB, Necchi A, Hussain S, Morales R (2016). Atezolizumab (atezo) in platinum (plat)-treated locally advanced/metastatic urothelial carcinoma (mUC): updated OS, safety and biomarkers from the Ph II IMvigor210 study. Ann Oncol.

[5] Patel MR, Ellerton JA, Infante JR, Agrawal M, Gordon MS, Aljumaliy R (2017). Avelumab in patients with metastatic urothelial carcinoma: pooled results from two cohorts of the phase 1b JAVELIN solid tumor trial. J Clin Oncol.

[6] Plimack ER, Bellmunt J, Gupta S, Berger R, Chow LQ, Juco J (2017). Safety and activity of pembrolizumab in patients with locally advanced or metastatic urothelial cancer (KEYNOTE-012): a non-randomised, open-label, phase 1b study. Lancet Oncol.

[7] Powles T, Eder JP, Fine GD, Braiteh FS, Loriot Y, Cruz C (2014). MPDL3280A (anti-PD-L1) treatment leads to clinical activity in metastatic bladder cancer. Nature.

[8] Powles T, O'Donnell PH, Massard C, Arkenau HT, Friedlander TW, Hoimes CJ (2017). Efficacy and safety of durvalumab in locally advanced or metastatic urothelial carcinoma: updated results from a phase 1/2 open-label study. JAMA Oncol.

[9] Rosenberg JE, Hoffman-Censits J, Powles T, van der Heijden MS, Balar AV, Necchi A (2016). Atezolizumab in patients with locally advanced and metastatic urothelial carcinoma who have progressed following treatment with platinum-based chemotherapy: a single-arm, multicentre, phase 2 trial. Lancet.

[10] Sharma P, Callahan MK, Bono P, Kim J, Spiliopoulou P, Calvo E (2016). Nivolumab monotherapy in recurrent metastatic urothelial carcinoma (CheckMate 032): a multicentre, open-label, two-stage, multi-arm, phase 1/2 trial. Lancet Oncol.

[11] Sharma P, Retz M, Siefker-Radtke A, Baron A, Necchi A, Bedke J (2017). Nivolumab in metastatic urothelial carcinoma after platinum therapy (CheckMate 275): a multicentre, single-arm, phase 2 trial. Lancet Oncol..

[12] Balar AV, Castellano D, O'Donnell PH, Grivas P, Vuky J, Powles T (2017). First-line pembrolizumab in cisplatin-ineligible patients with locally advanced and unresectable or metastatic urothelial cancer (KEYNOTE-052): a multicentre, single-arm, phase 2 study. Lancet Oncol..

[13] IMFINZI (durvalumab) [prescribing information]. AstraZeneca Pharmaceuticals LP, Wilmington; 2017.

[14] TECENTRIQ (atezolizumab) [prescribing information]. Genentech, Inc, San Francisco; 2016.

[15] OPDIVO (nivolumab) [prescribing information]. Bristol-Myers Squibb Company, Princeton; 2017.

[16] KEYTRUDA (pembrolizumab) [prescribing information]. Merck Sharp & Dohme Corp, White House Station, NJ; 2017.

[17] BAVENCIO (avelumab) [prescribing information]. EMD Serono, Inc, Rockland; 2017.

[18] OPDIVO (nivolumab) [summary of product characteristics]. Bristol-Myers Squibb Pharma EEIG, Uxbridge; 2015.

[19] KEYTRUDA (pembrolizumab) [summary of product characteristics]. Merck Sharp & Dohme Limited Hertfordshire; 2015.

[20] TECENTRIQ (atezolizumab) [summary of product characteristics]. Roche Registration Limited, Welwyn Garden City; 2017.

[21] Boyd ZS, Smith D, Baker B, Vennapusa B, Koeppen H, Kowanetz M (2016). Development of a PD-L1 companion diagnostic IHC assay (SP142) for atezolizumab. Cancer Immunol Res.

[22] Galsky MD, Retz M, Siefker-Radtke AO, Baron A, Necchi A, Bedke J (2016). Efficacy and safety of nivolumab monotherapy in patients with metastatic urothelial cancer (mUC) who have received prior treatment: results from the phase II CheckMate 275 study. Ann Oncol.

[23] Phillips T, Simmons P, Inzunza HD, Cogswell J, Novotny J, Taylor C (2015). Development of an automated PD-L1 immunohistochemistry (IHC) assay for non-small cell lung cancer. Appl Immunohistochem Mol Morphol.

[24] Ratcliffe MJ, Sharpe A, Rebelatto M, Scott M, Barker C, Scorer P (2016). A comparative study of PD-L1 diagnostic assays in squamous cell carcinoma of the head and neck (SCCHN). Ann Oncol.

[25] Rebelatto MC, Midha A, Mistry A, Sabalos C, Schechter N, Li X (2016). Development of a programmed cell death ligand-1 immunohistochemical assay validated for analysis of non-small cell lung cancer and head and neck squamous cell carcinoma. Diagn Pathol.

[26] Roach C, Zhang N, Corigliano E, Jansson M, Toland G, Ponto G (2016). Development of a companion diagnostic PD-L1 immunohistochemistry assay for pembrolizumab therapy in non-small-cell lung cancer. Appl Immunohistochem Mol Morphol.

[27] PD-L1 IHC 22C3 pharmDx [package insert]. Agilent Technologies Santa Clara; 2015.

[28] PD-L1 IHC 28-8 pharmDx [package insert]. Agilent Technologies, Santa Clara; 2015.

[29] VENTANA PD-L1 SP142 [package insert]. Ventana Medical Systems, Inc, Tucson; 2016.

[30] VENTANA PD-L1 SP263 [package insert]. Ventana Medical Systems, Inc, Tucson; 2017.

[31] Ratcliffe MJ, Sharpe A, Midha A, Barker C, Scott M, Scorer P (2017). Agreement between programmed cell death ligand-1 diagnostic assays across multiple protein expression cutoffs in non-small cell lung cancer. Clin Cancer Res.

[32] Buttner R, Gosney JR, Skov BG, Adam J, Motoi N, Bloom KJ (2017). Programmed death-ligand 1 immunohistochemistry testing: a review of analytical assays and clinical implementation in non-small-cell lung cancer. J Clin Oncol.

[33] Al-Masri H, Ratcliffe M, Sharpe A, Barker C, Scorer P, Scott M (2017). Concordance of tumour and immune cell staining with Ventana SP142, Ventana SP263, Dako 22C3 and Dako 28–8 PD-L1 tests across different cancer types. Virchows Arch.

[34] Schwamborn K, Knüchel R, Hartmann A, Baretton G, Lasitschka F, Schirmacher P (2017). Comparability of programmed death-ligand 1 (PD-L1) expression on tumor-infiltrating immune cells (IC) and tumor cells (TC) in advanced urothelial bladder cancer (UBC) using clinically relevant immunohistochemistry (IHC) assays. Ann Oncol.

[35] Feng Z, Schlichting M, Helwig C, Chand VK, Gelb A, Jin H (2017). Comparative study of two PD-L1 expression assays in patients with non-small cell lung cancer (NSCLC). J Clin Oncol.

[36] Dirix LY, Takacs I, Jerusalem G, Nikolinakos P, Arkenau HT, Forero-Torres A (2018). Avelumab, an anti-PD-L1 antibody, in patients with locally advanced or metastatic breast cancer: a phase 1b JAVELIN solid tumor study. Breast Cancer Res Treat.

[37] Clopper C, Pearson ES (1934). The use of confidence or fiducial limits illustrated in the case of the binomial. Biometrika.

[38] Hirsch FR, McElhinny A, Stanforth D, Ranger-Moore J, Jansson M, Kulangara K (2017). PD-L1 immunohistochemistry assays for lung cancer: results from phase 1 of the blueprint PD-L1 IHC assay comparison project. J Thorac Oncol.

[39] Ratcliffe MJ, Sharpe A, Midha A, Barker C, Scorer P, Walker J. A comparative study of PD-L1 diagnostic assays and the classification of patients as PD-L1 positive and PD-L1 negative, Presented at: Proceedings of the 107th Annual Meeting of the American Association for Cancer Research. New Orleans: American Association for Cancer Research; 2016. Abstract LB-094

[40] Hodgson A, Slodkowska E, Jungbluth A, Liu SK, Vesprini D, Enepekides D (2018). PD-L1 immunohistochemistry assay concordance in urothelial carcinoma of the bladder and hypopharyngeal squamous cell carcinoma. Am J Surg Pathol.

[41] Tretiakova M, Fulton R, Kocherginsky M, Long T, Ussakli C, Antic T (2018). Concordance study of PD-L1 expression in primary and metastatic bladder carcinomas: comparison of four commonly used antibodies and RNA expression. Mod Pathol.

