# Supplementary material for: Concordance among four commercially available, validated programmed cell death ligand-1 assays in urothelial carcinoma
Source: Diagn Pathol. 2019 Sep 2;14:99. doi: 10.1186/s13000-019-0873-6 (PMC6720992; doi:10.1186/s13000-019-0873-6)
Supplement: Supplementary file 1 — Non-urothelial bladder cancer samples included in the analysis (DOCX 14 kb) [file 13000_2019_873_MOESM1_ESM.docx]

| **Primary Diagnosis** | **N** |
| --- | --- |
| Adenocarcinoma | 1 |
| Invasive carcinoma | 1 |
| Squamous cell carcinoma | 7 |
| Sarcomatoid carcinoma | 1 |
| Small cell carcinoma | 1 |

Additional file 1 Non-urothelial bladder cancer samples included in the analysis
