# Supplementary figures and images for: Concordance among four commercially available, validated programmed cell death ligand-1 assays in urothelial carcinoma
Source: Diagn Pathol. 2019 Sep 2;14:99. doi: 10.1186/s13000-019-0873-6 (PMC6720992; doi:10.1186/s13000-019-0873-6)

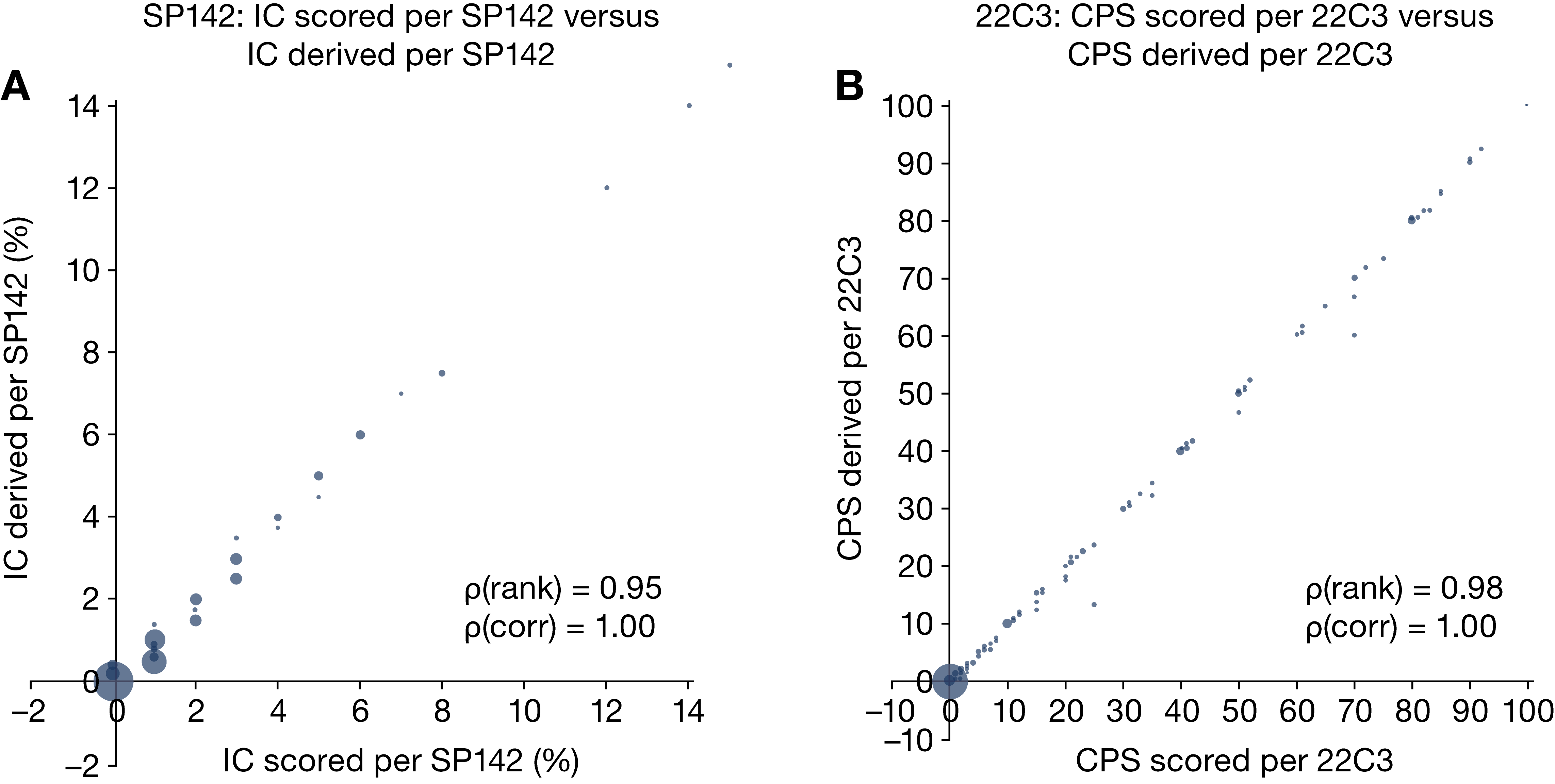

Supplement: Supplementary file 2 — Plots for derived versus scored staining for VENTANA SP142 immune cell scores (A) and PD-L1 IHC 22C3 pharmDx CPS (B). IC immune cells, CPS combined positive score (TIF 1582 kb) [file 13000_2019_873_MOESM2_ESM.tif]

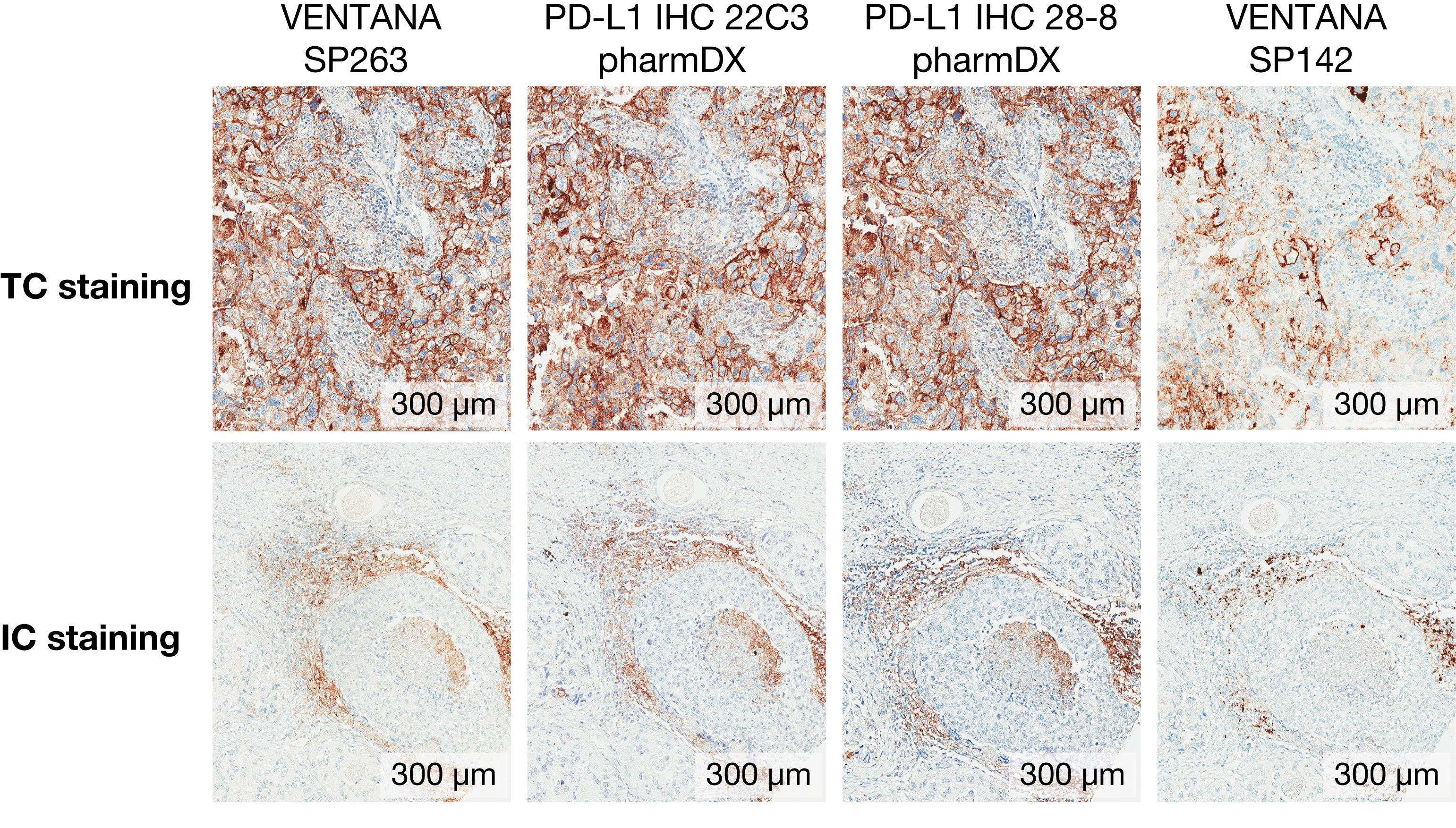

Supplement: Supplementary file 4 — PD-L1 staining for four PD-L1 assays. IC immune cells, TC tumor cells (TIF 22699 kb) [file 13000_2019_873_MOESM4_ESM.tif]
