# Supplementary material for: Concordance among four commercially available, validated programmed cell death ligand-1 assays in urothelial carcinoma
Source: Diagn Pathol. 2019 Sep 2;14:99. doi: 10.1186/s13000-019-0873-6 (PMC6720992; doi:10.1186/s13000-019-0873-6)
Supplement: Supplementary file 3 — PD-L1 proportion of samples above cutoff by assay, cutoff, and scored compartment. SP142 detects fewer TC PD-L1 positive cells than other PD-L1 assays. IC immune cells, PD-L1 programmed cell death ligand-1, TC tumor cells (DOCX 15 kb) [file 13000_2019_873_MOESM3_ESM.docx]

|  | **TC** | | | | **IC**  **(by IC area)** | | | | **IC**  **(by tumor area, as per SP142)** | | | |
| --- | --- | --- | --- | --- | --- | --- | --- | --- | --- | --- | --- | --- |
| **Cutoff** | **SP263** | **28-8** | **22C3** | **SP142** | **SP263** | **28-8** | **22C3** | **SP142** | **SP263** | **28-8** | **22C3** | **SP142** |
| **≥1%** | **40.9%** | **36.4%** | **37.0%** | **26.9%** | **63.9%** | **57.9%** | **59.4%** | **60.9%** | **32.2%** | **30.1%** | **31.6%** | **30.1%** |
| **≥5%** | **29.6%** | **29.3%** | **29.0%** | **21.2%** | **63.9%** | **57.9%** | **59.4%** | **60.6%** | **5.7%** | **4.8%** | **5.4%** | **5.7%** |
| **≥10%** | **27.2%** | **24.8%** | **24.8%** | **16.4%** | **62.1%** | **55.2%** | **56.4%** | **57.0%** | **1.5%** | **1.8%** | **1.8%** | **1.2%** |
| **≥25%** | **17.9%** | **15.2%** | **16.4%** | **6.3%** | **25.1%** | **21.5%** | **24.2%** | **22.7%** | **0.6%** | **0.6%** | **0.6%** | **0.3%** |
| **≥50%** | **11.9%** | **10.7%** | **11.3%** | **3.6%** | **13.4%** | **10.4%** | **11.3%** | **12.2%** | **0.0%** | **0.0%** | **0.0%** | **0.0%** |
