# Supplementary material for: Concordance among four commercially available, validated programmed cell death ligand-1 assays in urothelial carcinoma
Source: Diagn Pathol. 2019 Sep 2;14:99. doi: 10.1186/s13000-019-0873-6 (PMC6720992; doi:10.1186/s13000-019-0873-6)
Supplement: Supplementary file 5 — OPA, PPA, and NPA between PD-L1 assays in invasive UC samples (N = 255) (DOCX 16 kb) [file 13000_2019_873_MOESM5_ESM.docx]

Additional file 5 OPA, PPA, and NPA between PD-L1 assays in invasive UC samples (*N*=255)

| **Clinical algorithm** | **Comparator assay** | **VENTANA SP263 (TC/IC 25%) assay used as reference,**  **% agreement (95% CI)** ^a^ | | |
| --- | --- | --- | --- | --- |
|  |  | **OPA** | **PPA** | **NPA** |
| TC or IC_ICArea_ ≥25%  (VENTANA SP263) | PD-L1 IHC 22C3 pharmDX | 92.5% (89.3%) | 88.1% (81.5%) | 95.5% (91.6%) |
|  | PD-L1 IHC 28-8 pharmDX | 90.2% (86.6%) | 80.2% (72.5%) | 96.8% (93.3%) |
|  | VENTANA SP142 | 83.5% (79.2%) | 64.4% (55.8%) | 96.1% (92.5%) |
| CPS ≥1  (PD-L1 IHC 22C3 pharmDX) | PD-L1 IHC 22C3 pharmDX | 76.9% (72.1%) | 93.1% (87.4%) | 66.2% (59.4%) |
| CPS ≥10  (PD-L1 IHC 22C3 pharmDX) | PD-L1 IHC 22C3 pharmDX | 82.0% (77.5%) | 69.3% (60.9%) | 90.3% (85.4%) |
| TC ≥1%  (PD-L1 IHC 28-8 pharmDX) | PD-L1 IHC 28-8 pharmDX | 75.7% (70.9%) | 72.3% (64.0%) | 77.9% (71.7%) |
| IC_TumorArea_ ≥5%  (VENTANA SP142) | VENTANA SP142 | 66.3% (61.1%) | 15.8% (10.2%) | 99.4% (97.0%) |
